# Within-host Competition Does Not Select for Virulence in Malaria Parasites; Studies with *Plasmodium yoelii*

**DOI:** 10.1371/journal.ppat.1004628

**Published:** 2015-02-06

**Authors:** Hussein M. Abkallo, Julie-Anne Tangena, Jianxia Tang, Nobuyuki Kobayashi, Megumi Inoue, Augustin Zoungrana, Nick Colegrave, Richard Culleton

**Affiliations:** 1 Malaria Unit, Institute of Tropical Medicine, Nagasaki University, Nagasaki, Japan; 2 Laboratory of Molecular Biology of Infectious Agents, Graduate School of Biomedical Sciences, Nagasaki University, Nagasaki, Japan; 3 Laboratory of Entomology, Wageningen University, Wageningen, The Netherlands; 4 Institute of Evolutionary Biology, School of Biological Sciences, University of Edinburgh, Edinburgh, United Kingdom; Case Western Reserve University, UNITED STATES

## Abstract

In endemic areas with high transmission intensities, malaria infections are very often composed of multiple genetically distinct strains of malaria parasites. It has been hypothesised that this leads to intra-host competition, in which parasite strains compete for resources such as space and nutrients. This competition may have repercussions for the host, the parasite, and the vector in terms of disease severity, vector fitness, and parasite transmission potential and fitness. It has also been argued that within-host competition could lead to selection for more virulent parasites. Here we use the rodent malaria parasite *Plasmodium yoelii* to assess the consequences of mixed strain infections on disease severity and parasite fitness. Three isogenic strains with dramatically different growth rates (and hence virulence) were maintained in mice in single infections or in mixed strain infections with a genetically distinct strain. We compared the virulence (defined as harm to the mammalian host) of mixed strain infections with that of single infections, and assessed whether competition impacted on parasite fitness, assessed by transmission potential. We found that mixed infections were associated with a higher degree of disease severity and a prolonged infection time. In the mixed infections, the strain with the slower growth rate was often responsible for the competitive exclusion of the faster growing strain, presumably through host immune-mediated mechanisms. Importantly, and in contrast to previous work conducted with *Plasmodium chabaudi*, we found no correlation between parasite virulence and transmission potential to mosquitoes, suggesting that within-host competition would not drive the evolution of parasite virulence in *P. yoelii*.

## Author Summary

Malaria infections are very often composed of multiple strains of malaria parasites. It is thought that these strains may compete for resources such as space and nutrients within the host. Here we show that such “within-host competition” has repercussions for the virulence of the malaria infection, so that infections composed of multiple strains are more virulent in terms of disease severity, than single strain infections containing the constituent parasites. Following from this, it has been proposed that as such competition would favour those parasites with faster growth rates, then these parasites would be selected in nature when within-host competition is common. We show, however, that this is not necessarily the case, as parasites with faster growth rates in the mammalian host were no more successful at transmitting to mosquitoes than parasites with slower growth rates. These results show that a reassessment of our current understanding of the role of within-host competition in the selection of virulence in malaria parasites is required.

## Introduction

Malaria is caused by a diverse group of parasites composed of at least six species of the genus *Plasmodium*. Genetic diversity within these species is high, with multiple strains often co-infecting the same host, and is driven and maintained by mutation and through recombination between strains. Concomitant infection of hosts, both man and mosquitoes, with multiple species and/or strains is a common occurrence in endemic areas [1–3]. Such infections may result from the bites of multiply infected mosquitoes or from the bites of multiple mosquitoes harbouring different species or strains. Co-infecting species or strains interact during their life cycles and such interactions may lead to intra-host competition with repercussions for the host, the parasite, and the vector in terms of disease severity, vector fitness, and parasite transmission potential and fitness.

Within-host interactions between different parasite genotypes have been observed in both empirical human [4, 5] and rodent [6] malaria studies, and these have often been observed to result in modulations of parameters such as infection dynamics (suppression or enhancement of a particular strain or species in a mixed infection) and virulence (harm caused to the host). A series of experiments performed exclusively with strains of the rodent malaria parasite *Plasmodium chabaudi*, suggested that faster growing strains gained a competitive advantage over slower growing strains [7], in that they often dominated mixed strain infections in terms of proportional numbers of parasites, and sometimes competitively excluded the slower growing strain at some point during the infection. This has been interpreted as suggesting that within-host competition could lead to the selection of virulence within a parasite population.

The effects of competition between parasites on disease pathology is of particular relevance in malaria, as understanding the links between parasite genetic and disease severity will allow an understanding of how interventions, such as drugs and vaccines, that reduce parasite diversity will impact human health. There are conflicting theories as to how the presence of multiple strains (and/or species) of malaria parasites in an infection impact on disease. Reports from malaria endemic regions suggest that it is possible that disease pathology may be exacerbated by within-host competition [8] but also that it can result in decreased parasite burden and may protect against some clinical outcomes of disease [9–14].

Interactions between different *Plasmodium* strains or species concurrently infecting the same host (vertebrate or vector) may also influence the transmission dynamics of each species or strain, affecting their fitness, and driving the selection of those parasites that are good at competing. This has been observed in both human [3, 15–18] and rodent [19, 20] malaria infections. Laboratory experiments, field studies and mathematical modelling have been employed to describe the mechanisms driving the evolution of various phenotypes, including virulence. Virulence (defined as “harm to the host”) is often, but not always, linked to replication rate, with more virulent strains growing and replicating faster in the host than less virulent strains. Virulence differences can occur as a result of inherent genetic differences between strains, and/or through the influence of environmental factors, the most relevant of which to a parasite is the condition of the host [21]. It has been proposed that the evolution of virulence is driven by within-host competition between strains of malaria parasites in mixed infections [7, 22]. This theory is based on the idea that faster multiplying parasites out-compete others in a mixed infection and therefore transmit more successfully to the vector.

The consequences of within-host competition during the mosquito stage development of malaria parasites for both the vector and the parasite are very poorly understood. This is partially due to the fact that there is little understanding of how malaria parasite infection influences mosquito fitness. Some reports associate malaria parasite infection with decreased survival and reproduction of mosquitoes [23–29], others find no effect [30] while there is also evidence to suggest that that malaria infection increases longevity in mosquitoes due to a trade-off with decreasing reproduction [31]. These contradictions notwithstanding, many lab-based and field studies have convincingly established that *Plasmodium* causes pathological changes not only in their vertebrate hosts, but also in insect vectors. This is particularly evident during earlier stages of *Plasmodium* infection in the mosquito whereby ookinetes penetrate mosquito midgut epithelium (physical damage) and provoke physiological stress [32]. As a result, the mosquito launches an immune response to curb the infection, which is an energetically demanding process [33], hence reduced fitness. In addition, meta-analyses suggest that malaria parasites reduce mosquito fitness and survival [34]. Overall, *Plasmodium* infection is generally considered to be harmful to their mosquito hosts. What effect competition between parasite species and/or strains has on the pathology of the mosquito stages of malaria infections is largely unknown.

Here we use the rodent malaria parasite *Plasmodium yoelii* to explore the consequences of within-host competition on disease severity and parasite fitness (including transmission potential) between isogenic parasite strains with varying degrees of virulence.

## Results

### Mixed strain parasite infections are more virulent than single strain infections

Infection parameters for all single and mixed infections are summarised in Table 1. Of the three strains, only the virulent strain (17XL) causes death of mice in single strain infections. In mice infected with this strain, death occurs early in the infection, by day 5 post-infection. Neither of the avirulent strains (17XNL and CU) or the intermediately virulent strain (17X1.1pp) cause host mortality at any time, and infections self-clear within 30 days.

**Table 1:** Infection parameters for single and mixed strain infections.

**Strain**	**CU**	**17XNL**	**17X1.1pp**	**17XL**	**CU + 17XNL**	**CU+17X1.1pp**	**CU+17XL**
**RBC Invasion Preference**	Reticulocytes	Reticulocytes	Reticulocytes, subset of Normocytes	Reticulocytes and Normocytes	—	—	—
**Peak Parasitaemia (day pi)**	39.8% ± 1.2 (18–19)	30.8% ± 1.3 (18, 20)	50.6% ± 2.4 (17)	91.9% ± 0.9 (5)	36.7% ± 0.9 (14–17)	50.3% ± 2.1 (14–16)	83.1% ± 1.9 (5)
**Cumulative Parasitaemia**	379.20	337.30	619.80	129.80	407.40	736.70	109.30
**Mortality (day pi)**	0%	0%	0%	100% (5)	0%	75% (26, 28, 30)	100% (5)
**Max. Weight Loss, g (day pi)**	3.15 ± 0.6 (20)	2.09 ± 0.2 (14, 20)	4.05 ± 0.2 (19, 20)	1.68 ± 0.3 (5)	3.36 ± 0.3 (19, 21, 23)	6.03 ± 0.6 (17, 26, 28, 29)	0.75 ± 0.4 (5)
**Min. RBC Count, RBC/mL (day pi)**	1.70×10^9^ ± 1.34×10^8^ (18, 20–22)	2.06×10^9^ ± 9.10×10^7^ (15,17)	1.49×10^9^ ± 1.29×10^8^ (14, 19)	2.96×10^9^ ± 1.52×10^8^ (5)	1.56×10^9^ ± 3.23×10^8^ (17, 22, 23)	9.75×10^8^ ± 1.16×10^8^ (22, 26, 27)	2.73×10^9^ ± 1.54×10^8^ (5)

Peak parasitaemia; Mean (±SEM) of highest parasitaemia, Cumulative parasitaemia; Mean of area under curve of the parasitaemia curve in single and mixed infections. Maximum Weight loss is the Mean (±SEM) of maximum weight lost by the infected mice. Minimum RBC count is the Mean (±SEM) of least Red Blood Cell number per mL of blood. Days post infections are given in parentheses. Data were generated from groups of 4 mice, and are representative of two independent repeat experiments.

To analyse time course data we fitted general linear mixed models with Treatment and Time and their interaction as fixed factors, and Mouse as a random factor nested within treatment. To account for possible autocorrelation of errors through time, we compared the fit of models with and without an autocorrelation term [35]. Models were fitted using REML, and compared using Likelihood ratio tests. In cases where the autocorrelation term significantly improved the fit of the model it was retained for subsequent analysis of the fixed effects. The significance of fixed effects was then determined by fitting models with and without the term of interest using Maximum Likelihood, and comparing the fit of these models with a Likelihood ratio test. Parasitaemia values were log transformed prior to analysis to meet assumptions of homogeneity of variance whilst other response variables were analysed on the measured scale. All analyses were carried out using the LME function in R [36]. Time courses for infections involving the virulent strain were only analysed up to day 5, after which point all mice had died.

**Figure 1:**
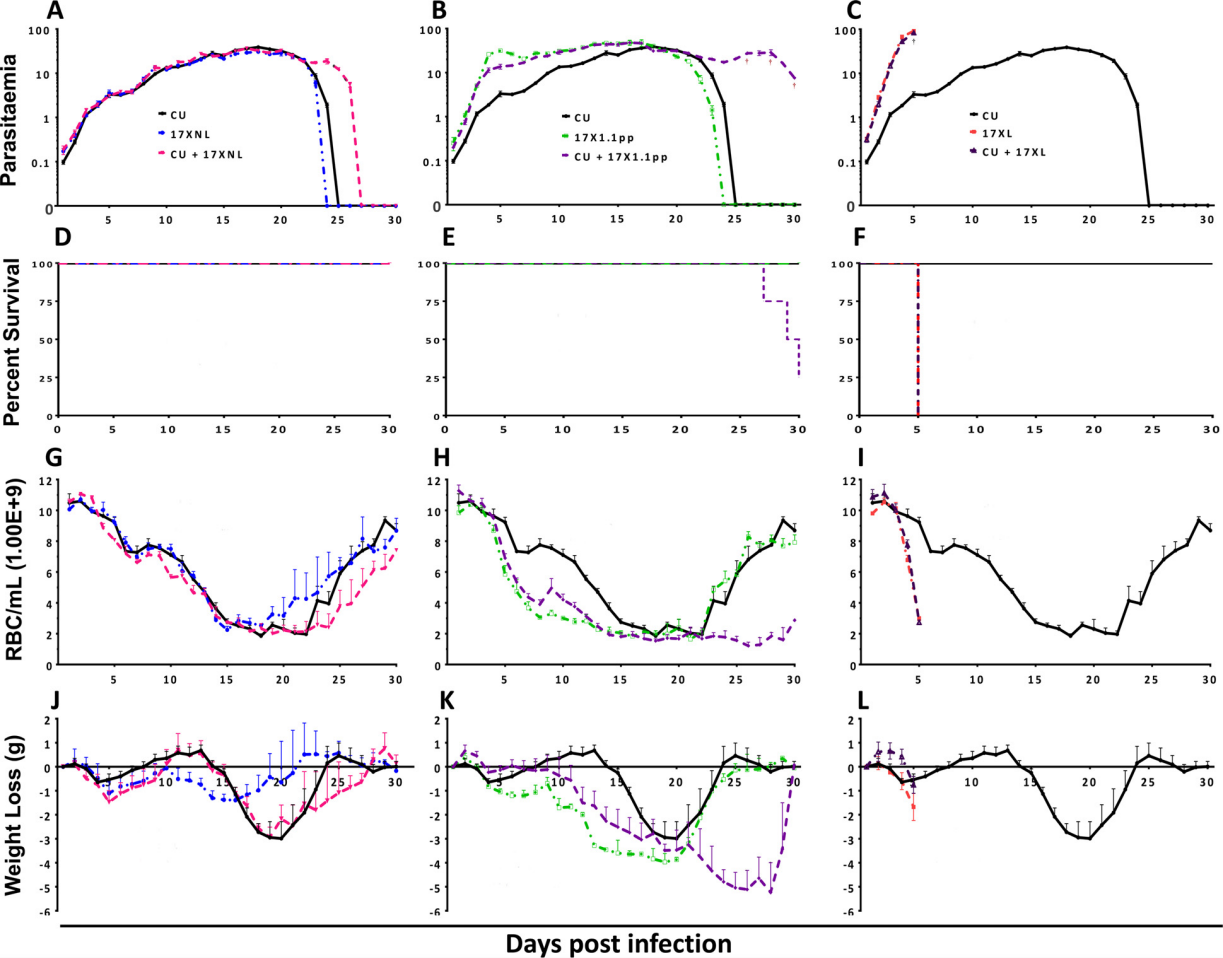
Parasitaemia (Panels A-C), percentage survival (D-F), red blood cell density (G-I) and weight change (J-L) of mice infected with avirulent (CU and 17XNL), intermediately virulent (17X1.1pp) and virulent (17XL) strains of *Plasmodium yoelii yoelii* in single or in mixed infections. Data points indicate the mean value for 4 mice per experimental group. † indicates the time points at which individual mice died. Data are representative of two independent experiments.

Both avirulent/avirulent (CU+17XNL) and avirulent/intermediately virulent (CU+17X1.1pp) strain mixed infections were characterised by protracted parasitaemia and prolonged chronicity of disease compared to their constituent strains growing in single strain infections. The mixed strain infections resulted in higher parasitaemia than either of the constituent strains in single infections late on in the infection (treatment by time interaction term, L = 306.28, df = 50, P < 0.0001, and L = 579.48, df = 50, P < 0.0001, Fig. 1, Panels A and B). For the avirulent/virulent (CU+17XL) mixed strain infections, the course of infection followed that of the most virulent constituent strain (Fig. 1 Panel C, treatment by time interaction term, L = 111.69, DF = 8, P < 0.001). Strikingly, infection with the avirulent/intermediately virulent mixture resulted in 75% host mortality infection late in the infection (Log-rank test, χ^2^ = 8.165, df = 2, *p* = 0.0169, Fig. 1 Panel E), and this was associated with the inability to clear parasites from the blood.

In order to compare the virulence (here defined as pathological harm to the host) of mixed strain infections to that of single strain infections, we measured the red blood cell (RBC) density, and weight of mice daily throughout the course of the infections (Fig. 1, panels G-I and J-L, respectively), sampling ceasing when parasites were no longer visible in the blood by microscopy, or when mice succumbed to infection.

Mixed infections of avirulent/avirulent (CU + 17XNL) resulted in lower RBC density and greater weight loss compared to either of the constituent strains in single infections during the latter stages of the infection (Fig. 1, Panels G, L = 97.90, DF = 86, P = 0.0004 and J, L = 131.38, DF = 86, P < 0.0001), a phenomenon consistent with the prolonged chronicity of the mixed infection parasitaemia. In the avirulent/intermediately virulent (CU + 17X1.1pp) mixed infection, this effect was more pronounced, with dramatic and significantly lower RBC count (Fig. 1, panel H, L = 228.75 DF = 86, P < 0.0001) and significantly greater weight loss (Fig. 1, panel K, L = 171.70 DF = 86, P < 0.0001) compared to single strain infections, This weight loss and reduction in RBC density occurred in the latter part of the infection, reflecting the significantly higher parasitaemia of the mixed infection group during this period. Single infections of the most virulent strain (17XL) result in acute anaemia and dramatic weight loss early in the infection, and this pattern is also observed in the mixed strain infection containing this strain plus an avirulent strain (CU + 17XL) (Fig. 1, Panels I and L).

### Mixed strain infections do not generally result in significantly different numbers of infected mosquitoes or oocyst burdens than their constituent single strain infections

In order to determine whether mixed strain infections infect more mosquitoes and result in higher oocyst burdens than single strain infections, we allowed *A. stephensi* mosquitoes to feed on mice with single or mixed strain infections. As transmissibility varies dramatically throughout an infection, we allowed mosquitoes to feed at two separate time points; on day 3 post-infection, when the oocyst conversion rate (OCR, the number of oocysts produced per gametocyte) is at its highest in *P. yoelii* infections (R. Culleton, unpublished observations), and on day 4 post-inoculation when OCR is lower.

We observed no significant differences in the numbers of gametocytes produced in single strain infections compared to mixed strain infections, with the exception of the mixed infection composed of the avirulent CU and the intermediately virulent 17X1.1pp on day 3 pi, which contained significantly fewer gametocytes than the single 17X1.1pp infection (S1 Fig.).

**Figure 2:**
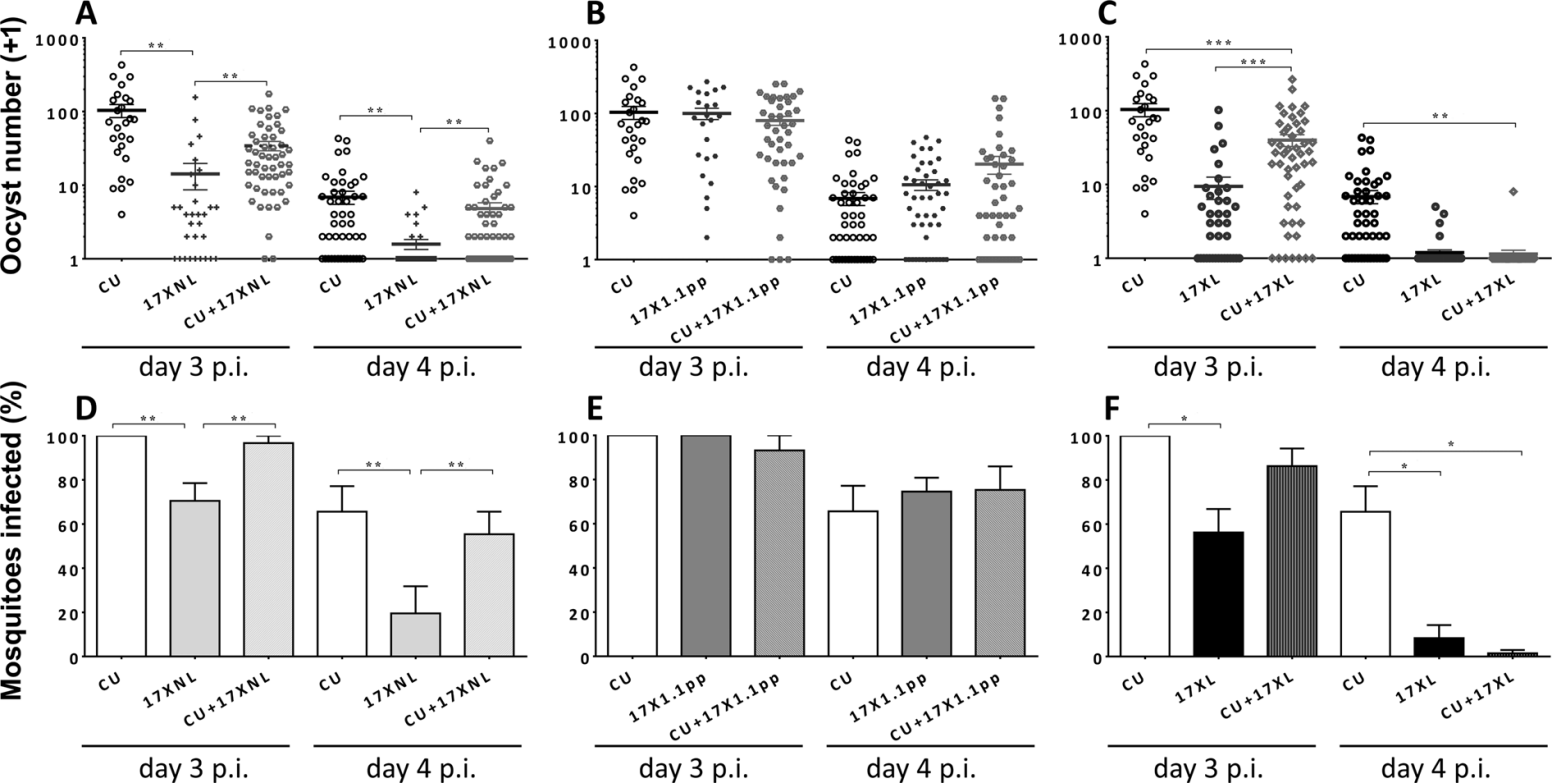
Transmission success of strains of *Plasmodium yoelii yoelii* in single and mixed strain infections. **Panels A, B** and **C** show the numbers of oocysts per mosquito fed on mice infected with CU (avirulent), 17XNL (avirulent), 17X1.1pp (intermediately virulent) and 17XL (virulent) either in single or in mixed infections on days 3 and 4 post-inoculation. Error bars indicate the standard error of the mean. Twenty mosquitoes were allowed to feed on each mouse, and those that were gravid after 7 days were considered blood-fed and included in the analysis. **Panels D, E** and **F**; the mean percentage of mosquitoes infected with at least one oocyst following feeding on each of the strains in single or mixed infections. Error bars indicate the standard error of the mean for mosquitoes fed on groups of 4 mice. Data are representative of two independent experiments. * = P < 0.05, ** = P < 0.005, *** = P < 0.001

All infections, regardless of virulence or whether mixed or single, resulted in a lower percentage of mosquitoes infected and lower oocyst burdens following feeding on day 4 pi compared to day 3 pi (Fig. 2). For the single infections, the greatest percentages of infected mosquitoes (mean, n = 4 mice) were found in those that had fed on CU (100%) or 17X1.1pp (100%), followed by those fed on 17XNL (70.5%) and 17XL (56.2%). The highest mean day 3 pi oocyst burdens were recorded for 17X1.1pp (105 oocysts per infected mosquito, p.i.m), followed by CU (96 oocysts p.i.m), 17XNL (17 oocysts p.i.m), and 17XL (10 oocysts p.i.m). This pattern was similar on day 4 pi, when the highest percentage of infected mosquitoes was achieved by 17X1.1pp (74.5%), followed by CU (65.6%), 17XNL (19.5%) and 17XL (8.3%), with associated mean oocyst burdens of 13, 8, 3, and 2 oocysts p.i.m, respectively. There was, therefore, no positive correlation between virulence and transmission potential.

To test for differences in transmission potential (defined as the percentage of mosquitoes carrying one or more oocyst following blood feeding on infected mice) between mixed and single infections, we fitted a generalised linear mixed model, with treatment and day and their interaction as fixed factors, and mouse as a random factor to account for the repeated measures on each mouse. Since infection is a binary trait, we fitted a binomial error structure. Models were fitted using the glmer function in R with Likelihood ratio tests used to compare models with different fixed effects.

In mixed infections containing two avirulent (CU + 17XNL), There was no significant interaction between day and treatment, (L = 2.231, df = 2, P > 0.05), whilst treatment affected the proportion infected on both days (L = 12.369, df = 2, P = 0.002, with 17XNL infecting fewer mosquitos than either CU or the mixture (Fig. 2, Panel D).

For the mixed infection containing one avirulent and one intermediately virulent strain (CU + 17X1.1pp), again, there was no indication of an interaction (L = 4.75, df = 2, P > 0.05). Infection was lower on day 4, than on day 3 (L = 30.545, df = 1, P < 0.001), but there was no effect of treatment (L = 0.2656, df = 2, p > 0.05, Fig. 2, Panel E).

When mosquitoes fed on mice infected with the avirulent/virulent (CU + 17XL) mixed infection, the effect of treatment on infection depended on day (L = 6.681, df = 2, P < 0.05), with CU having higher infectivity than both other treatments on day 4, and than 17XL on day 3, (Fig. 2, Panel F).

In summary, mixed infections did not infect significantly different percentages of mosquitoes than one of the constituent strains (CU, the most successful transmitter), with the exception of the CU+17XL infection when mosquitoes fed on day 4 of the infection. In this case, there were significantly fewer mosquitoes infected than in the CU single infection.

To analyse oocyst number, we used a general linear mixed model with day and treatment as fixed factors, and mouse as a random factor. Data was logged prior to analysis to meet the assumptions of homogeneity of variance.

In mixed infections containing two avirulent (CU + 17XNL), there was a significant effect of treatment on oocyst numbers (treatment main effect, F_2,9_ = 13.71, p = 0.002), with CU producing more oocysts that the mixture and 17XNL producing less. There were fewer oocysts on day 4 than day 3 (F_1,9_ = 78.01, p < 0.001), but the effect of treatment was consistent on both days (F_2,9_ = 0.781, p = 0.4868). Post-hoc tests revealed that the mixed infection produced significantly more oocysts than 17XNL (P = 0.0031), but oocyst numbers did not differ significantly from CU in a single infection (Fig. 2, Panel A).

In the mixed infection composed of an avirulent and an intermediately virulent parasite (CU+17X1.1pp), there were no significant differences in the numbers of oocysts produced by the mixed infection compared to the constituent strain single infections (Fig. 2, Panel B).

When an avirulent and a virulent parasite strain co-infect a host (CU + 17XL), thee numbers of oocysts produced in mosquitoes fed on single strain and mixed infections is significantly different (treatment main effect, F_2,9_ = 18.28, p < 0.0001), with CU producing more oocysts than the mixture and 17XL producing less. There were fewer oocysts on day 4 than day 3 (F_1,9_ = 52.14, p < 0.001), but the effect of treatment was consistent on both days (F_2,9_ = 3.42, p = 0.0787). Post-hoc tests revealed that CU differs significantly from the mixture (P = 0.003), but for 17XL the difference is marginally non-significant (Fig. 2, Panel C).

### Parasite virulence and competitive ability of blood stage parasites are not linked in mixed infections of *P. yoelii*

**Figure 3:**
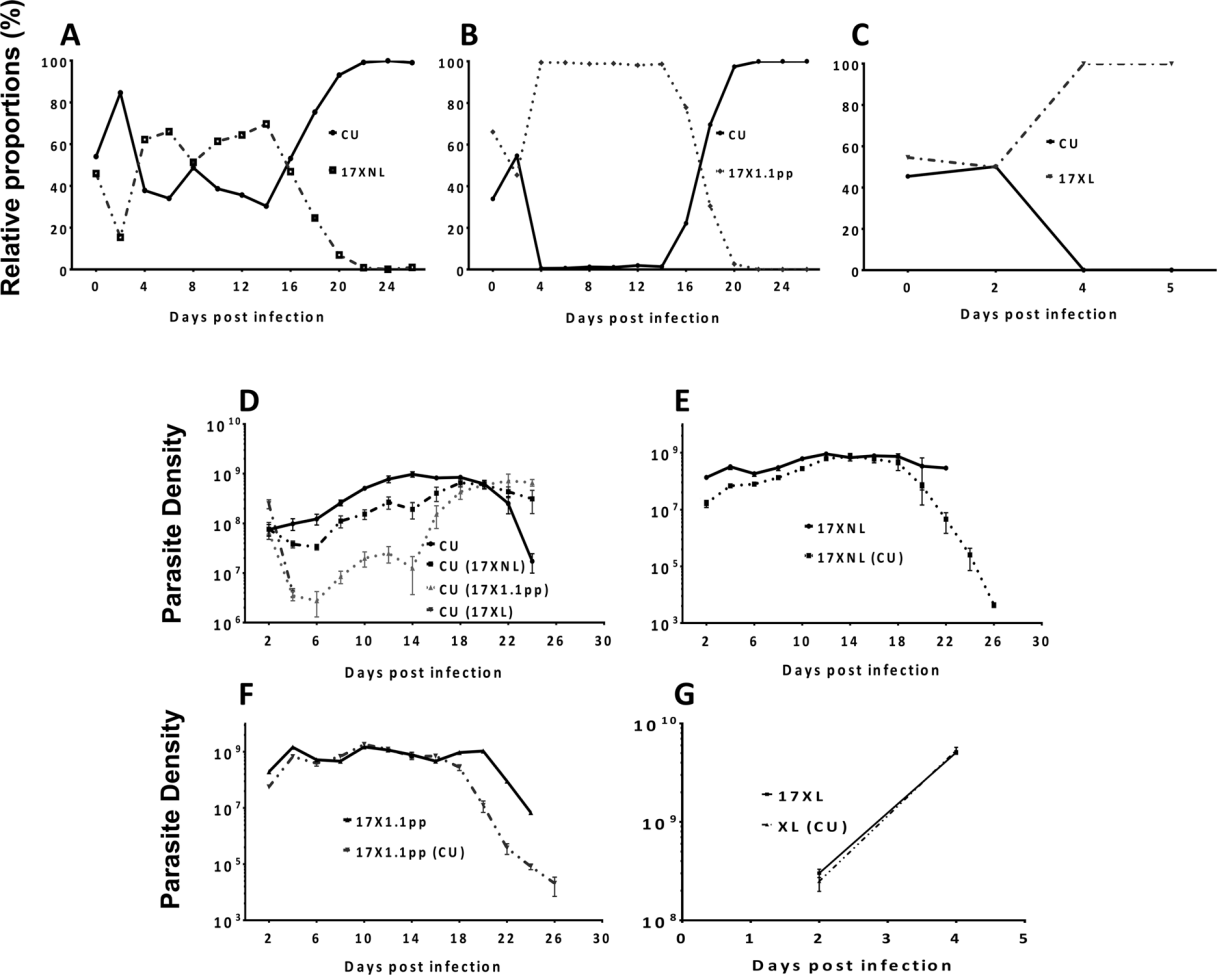
The relative proportions of strains of *Plasmodium yoelii yoelii* in mixed strain infections throughout the course of infections in mice (Panels A-C), and the parasite densities of the strains in single and mixed infections (Panels D-G). **Panels A, B** and **C** show the relative proportions of the avirulent CU strain in combination with the avirulent 17XNL strain, the intermediately virulent 17X1.1pp and the virulent strain 17XL respectively. Individual data points are mean proportions of 4 mice per group. The parasite density (number of asexual stage parasites per mL of blood) of the avirulent strain CU in a single infection is compared to the parasite density of this strain when in competition with the other strains in **Panel D**; that of avirulent 17XNL in a single infection or in competition with CU in **Panel E**; the intermediately virulent 17X1.1pp in a single infection or in competition with CU in **Panel F**; the virulent 17XL in a single infection or in competition with CU in **Panel G**. Strains in brackets indicate the competing strain. Error bars indicate the standard error of the mean of 4 mice per group. Data are representative of two independent experiments.

We measured the relative proportions of each of the strains within mixed infections by strain-specific qPCR every day throughout the course of the infection. In the mixed infection composed of the two avirulent strains CU and 17XNL, the proportions of the two strains fluctuate between 65% and 35% during the first 16 days of the infection, with 17XNL dominating for the majority of this period. From day 16 pi onwards, however, the proportion of 17XNL relative to CU drops daily until finally, at day 22 pi, there is complete competitive exclusion of 17XNL by CU (Fig. 3, panel A). In the mixed infection containing avirulent (CU) and intermediately virulent (17X1.1pp) parasites, the intermediately virulent strain completely dominates the infection from day 4 pi until day 14 pi, during which period no avirulent parasites could be detected. However, this situation is dramatically reversed from day 16 pi, when the avirulent parasite re-emerges, and completely dominates the infection from day 20 until the infection is cleared, completely competitively excluding the intermediately virulent strain (Fig. 3, panel B). In the case of the mixed infection composed of a virulent (17XL) and an avirulent (CU) parasite, the virulent parasite completely dominates the infection from day 4 pi, competitively excluding the avirulent parasite, as all mice die due to the infection on day 5 pi (Fig. 3, panel C).

We next compared the numbers of parasites produced by each strain (in terms of parasite density, defined as the number of parasites per mL of blood at a particular time-point) throughout the course of single infections, with the numbers produced when in competition with another strain. The avirulent strain CU is competitively supressed by all strains during the first 20 days of the infection, with competitive suppression strongest when in competition with the virulent strain (17XL) (mice die at day 5 pi), followed by the intermediate strain (17X1.1pp) and suppression mildest when in competition with the avirulent strain (17XNL). When in competition with the avirulent (17XNL) and the intermediately virulent (17X1.1pp) strains, competitive suppression ceases at day 20 pi, and competitive release occurs, with CU parasite densities reaching higher levels than in single infections (Fig. 3, Panel D). Thus, over the course of an infection in which CU is mixed with a strain of the same or higher virulence, both competitive suppression and facilitation occur.

The avirulent parasite 17XNL is not supressed in competition with CU compared to growth in single infections during the first 16 days of the infection, after which time it suffers competitive suppression (Fig. 3, panel E). A similar trend is seen with the intermediately virulent 17X1.1pp strain, which is completely unaffected by the presence of CU in mixed infections up to day 16, from which point on it suffers from competitive suppression (Fig. 3, panel F). The virulent 17XL strain is completely unaffected by the presence of an avirulent competitor (CU) in a mixed infection throughout the 5 days during which mice survive (Fig. 3, panel G).

### Parasite virulence is not linked to the transmission capacity of strains in single infections or in mixed infections

We measured the relative proportion of each of the strains in mixed infections in mosquitoes using qPCR on DNA extracted from mosquitoes with known numbers of oocysts, and compared the adjusted number of oocysts per strain (total number of oocysts multiplied by the frequency of the strain measured by qPCR) to the numbers produced in single infections. This analysis was performed on oocyst DNA extracted from mosquitoes fed on mice at days 3 and 4 pi.

We fitted a general linear mixed model, with infection treatment and day fitted as fixed factors, and mouse fitted as a random factor nested within treatment. Adjusted oocyst number was log transformed to meet homogeneity of variance assumptions. For the mixed infection containing the virulent parasite 17XL, as there was very little transmission on day 4, we were unable to use a general linear model, and t-tests were used in its place.

**Figure 4:**
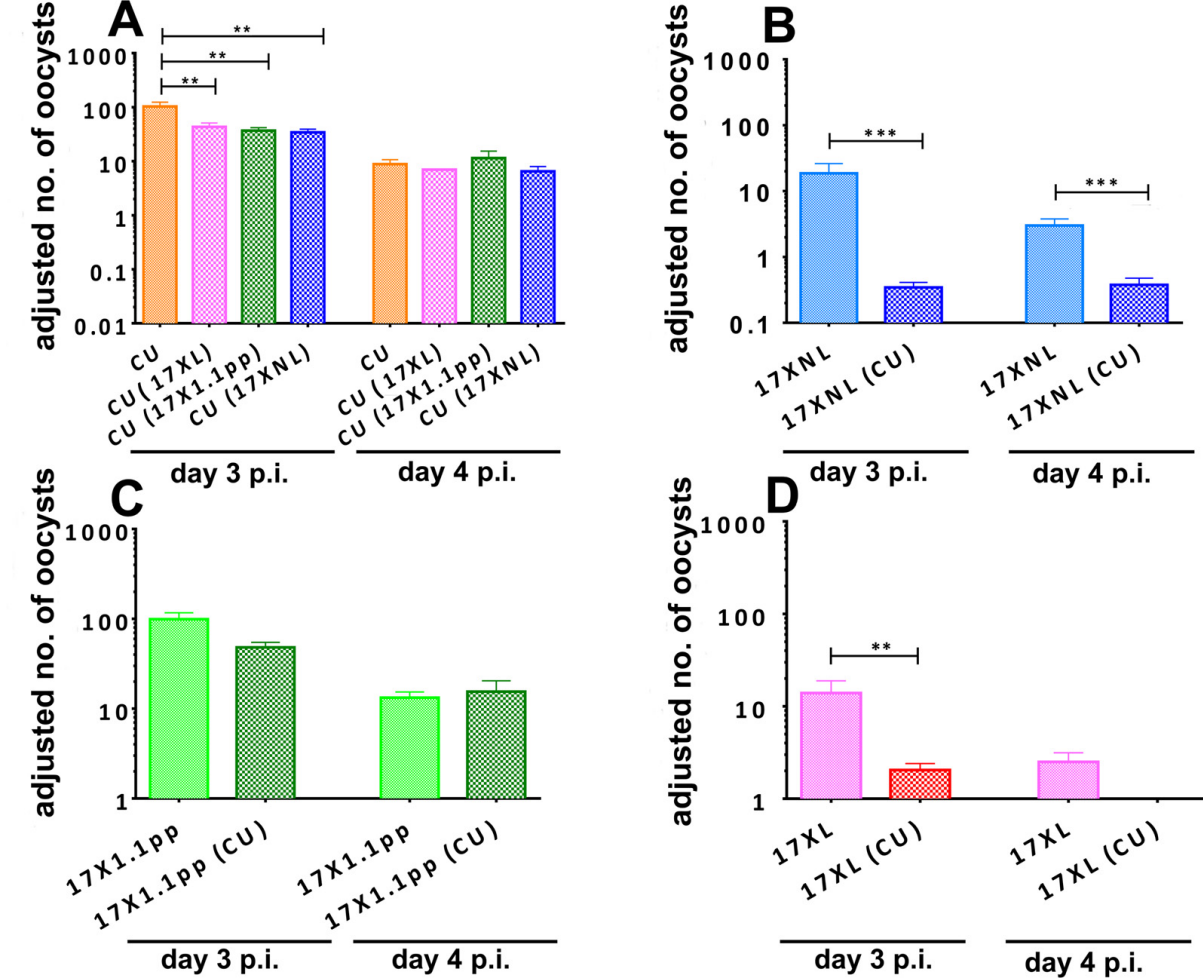
Adjusted mean number of oocysts per infected mosquito of strains of *Plasmodium yoelii yoelii* derived from mosquitoes fed on mice harbouring single strain or mixed strain infections on days 3 and 4 post-inoculation. Oocyst numbers are given for strain CU when in competition with all other strains (**Panel A**), for 17XNL in competition with CU (**B**), for 17X1.1pp in competition with CU (**C**) and for 17XL in competition with CU (**D**). Error bars indicate the standard error of the mean for 4 mice per group, and data are representative of two independent experiments. * = P < 0.05, ** = P < 0.005, *** = P < 0.001

The avirulent clone CU was significantly less successful at transmitting to mosquitoes in mixed infections with all competing strains on day 3 pi (F_3,12_ = 7.78, P = 0.0038), when transmission capacity is at its peak in the *P. yoelii* / *Mus musculus* / *Anopheles stephensi* malaria system. The effect of infection treatment differed for the two days (Treatment*Day interaction, F_3,12_ = 3.68, P < 0.0433) with CU not producing significantly differing numbers of oocysts in single compared to mixed infections on day 4 (Fig. 4, panel A). Importantly, the virulence of the competing clone had no effect on the degree of competitive suppression of transmission potential.

The transmission of the avirulent parasite 17XNL was severely and significantly reduced in mixed infections compared to single infections on both days 3 and 4 (F_1,9_ = 30.48, P = 0.0004, Fig. 4, panel B). The intermediately virulent strain 17X1.1pp suffered a less drastic and not statistically significant reduction in transmission success through competition with the avirulent strain CU on day 3 pi and there was no difference on day 4 (Fig. 4, panel C). Finally, the transmission of virulent strain 17XL to mosquitoes was severely and significantly reduced in the presence of an avirulent competitor on day 3 pi (Student’s two-tailed t-test, t = 3.282, df = 64, P = 0.0017), and completely abrogated on day 4 pi (Fig. 4, panel D).

**Figure 5:**
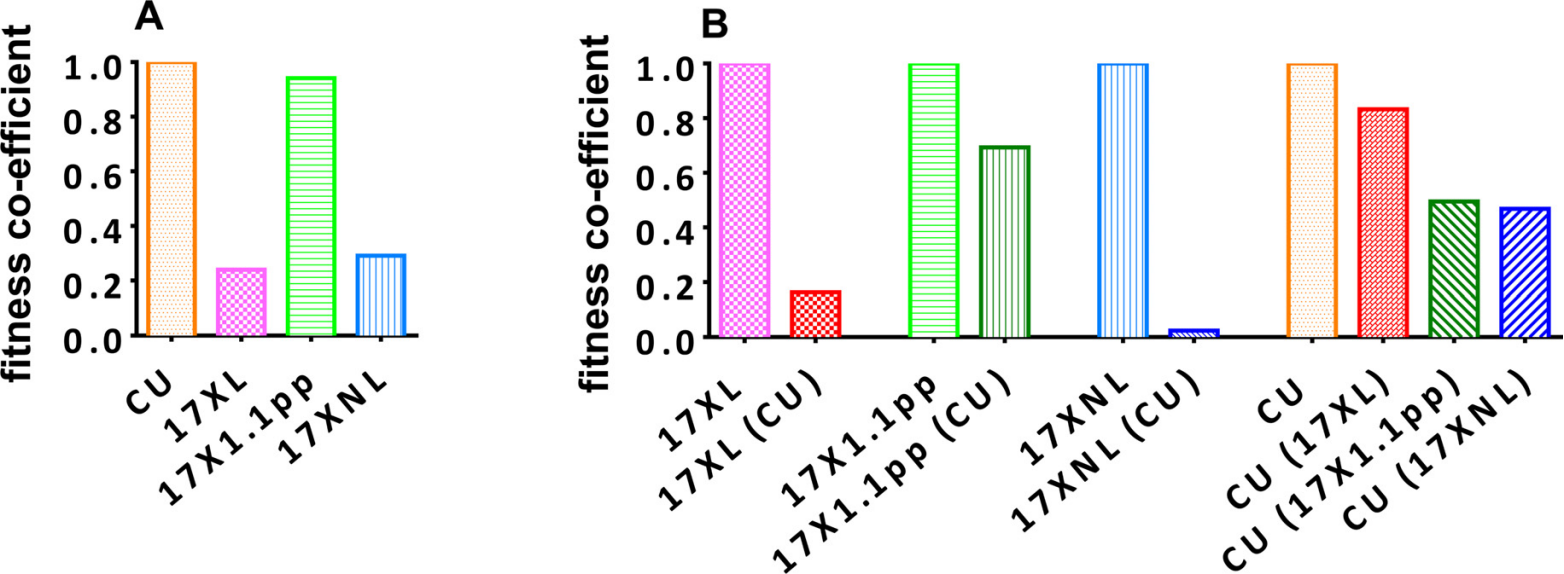
Relative fitness of avirulent (CU and 17XNL), intermediately virulent (17X1.1pp) and virulent (17XL) strains of *Plasmodium yoelii yoelii* in single (Panel A) or in mixed (B) infections. Fitness coefficients were derived from the numbers of oocysts produced by each clone in mixed and single infections. For single strain infections (**A**), the fitness of 17XNL, 17XL and 17X1.1pp are given relative to that of the fittest strain, CU. In mixed infections, the fitness of each clone in the presence of a competitor or competitors is given relative to its fitness in a single strain infection (**B**).

Using the oocyst data and relative proportions of each strain described above, we determined a fitness coefficient, reflecting the relative contribution of each strain to the products of fertilization in the mosquito midgut (i.e. oocysts) for each parasite strain either in single infections (Fig. 5, panel A), or in competition with the other strains (Fig. 5, panel B). The CU strain exhibits the highest fitness in single infections, followed by the intermediately virulent 17X1.1pp, the avirulent 17XNL, and finally the virulent 17XL. All strains are negatively affected by competition, with 17XL and 17XNL particularly severely compromised when in competition with CU. The CU strain is least affected by the presence of 17XL followed by 17X1.1pp and 17XNL.

In order to test whether infections with avirulent parasite are infectious to mosquitoes during the latter stages of the infection, we allowed mosquitoes to feed on mice infected with 17XNL on day 18. We found that these mosquitoes were infected with oocysts following feeding, and so transmission is possible during the latter stages of infection, at least with this strain.

Finally, we assessed whether the proportion of strains measured in oocysts was representative of the proportion of strains inoculated into mice during mosquito feeding. We found a good correlation between the proportions of strains in oocysts, and the proportions in the blood of mice in infections resulting from inoculation of sporozoites from tested mosquitoes (S2 Fig.).

## Discussion

**The consequences of within-host competition on disease severity in the mammalian host** Mixed strain malaria parasite infections, in which multiple genetically distinct parasites co-infect the same host, are common in nature, and are probably more common than single-clone infections, certainly in regions with relatively high transmission rates [4, 10, 37, 38, 39, 40, 41, 42, 43]. It is becoming increasing clear that malaria parasites co-infecting the same host interact with each other [43–45]. The consequences of these interactions for disease severity have been the subject of a limited number of field studies, some of which report that disease severity increases with increasing parasite genetic diversity within infections [46–48]. The general consensus from these studies is that co-infections result in competition between strains, and this competition can lead to increased virulence of co-infections compared with single clone infections [22].

Our results indicate that simultaneous inoculation with two genetically distinct strains of *P. yoelii* result in infections that are more virulent than single-clone infections composed of either of the co-infecting strains. Virulence in this case was measured by assessing host weight loss, RBC density and parasitaemia. This was true when the co-infections were composed of two avirulent strains or one avirulent and one intermediately virulent strain. When co-infections contained a very virulent strain and an avirulent strain, then the virulence of the co-infection was not significantly different from the virulence of the infection caused by the virulent strain. In this case, however, the virulent strain causes very severe disease, and host death within five days, making measurement of any possible increased virulence in a co-infection very difficult. Co-infections always resulted in infections that were either more virulent than each of the constituent strains, or which were as virulent as the most virulent of the constituent strains; we did not observe any protective effects of co-infection on host disease severity.

Our results show that in experiments with the rodent malaria parasite *P. yoelii*, mixed infections cause more harm to the host than single infections, a phenomenon also observed in natural human infections with *P. falciparum* [46–48]. If we extrapolate from these experiments and assume that mixed strain infections are, in general, more harmful than single clone infections, then a case may be made for the implementation of malaria control measures that aim to reduce parasite genetic diversity. Of course, most existing interventions such as drug treatment and anti-mosquito measures do exactly this; reduce parasite genetic diversity by reducing parasite prevalence rates. Extrapolation from rodent malaria parasites comes, however, with the usual caveats, and it should be mentioned that many field reports do not find any correlation between parasite genetic diversity (most commonly measured by the “multiplicity of infection” (MOI) index), and disease severity [49–54], and some find a negative correlation [55, 56]. It may also be the case that many field-based studies consider chronic malaria infections, whereas acute infections are considered in rodent malaria studies. There are also, of course, problems and difficulties with the interpretation of field-based surveys that do not apply to laboratory based experiments in which conditions can be carefully controlled and confounding factors minimised.

### Within-host competition and the evolution of virulence

It has been argued, on the basis of numerous experiments performed exclusively with *P. chabaudi*, that within-host competition leads to the selection of virulent malaria parasites [7, 22, 57–60]. This theory relies on the assumption that the “virulent” parasite (typically, the one with the fastest growth rate) outcompetes the less virulent parasites in the mammalian host, and then, crucially, is more successful at transmitting to mosquitoes, and subsequently into another mammalian host, than the less virulent parasites. Our results do not support this assumption. Firstly, the most virulent strain in a mixed infection does not always out-compete the least virulent. In the case of a mixed infection between an intermediately virulent and an avirulent parasite, it was ultimately the avirulent parasite that was responsible for the competitive exclusion of the intermediate virulence parasite. We hypothesise that the strain that dominates the infection during the acute phase (the first 7 days of the infection), is subsequently targeted by a stronger strain-specific immune response than the competing strain, leading to the competitive release of the less virulent clone later on in the infection. Crucially, we found that *P. y. yoelii* infections are infectious to mosquitoes during the latter stages of such infections, following the competitive exclusion of the most virulent strain. In the case of a mixed infection with a highly virulent strain, the avirulent strain was competitively excluded by the fifth day of the infection, at which point the death of the host occurred, effectively restricting the transmission of both the virulent and avirulent parasites to the first five days of the infection.

Secondly, we found no correlation between the virulence of a parasite and its transmission ability in single infections, with the avirulent strain (CU) resulting in the highest proportion of mosquitoes infected, and the highest number of oocysts per infected mosquitoes, than any of the other strains. Based on the transmission success of the clones in single infections, a fitness co-infection was derived which reflects the transmissibility of the strains on days three and four post-infection when transmissibility is at its highest in *P. y. yoelii*. This revealed that CU (avirulent) had the highest fitness in single infections, followed by 17X1.1pp (intermediately virulent), 17XNL (avirulent), and finally, the highly virulent 17XL. Furthermore, these relative finesses were calculated for only days three and four of the infections, and, as we found that transmission to mosquitoes was successful during the chronic phase of infection on the day on which it was tested, it is likely that the true relative fitness of the virulent 17XL is much lower than our estimates, as it kills the host on the fifth day of infection.

Thirdly, there was no correlation between a strain’s virulence and the relative fitness cost of competition with another strain. For example, the relative fitness of the highly virulent strain 17XL in mixed infection with the avirulent strain CU was ~20% of its fitness in a single infection, whilst the relative fitness of the intermediately virulent 17X1.1pp in a mixed infection with CU was ~70% of its fitness in a single infection. Of all the strains, the avirulent 17XNL suffered the largest cost of competition with the avirulent CU strain, followed by the virulent 17XL, and the intermediately virulent 17X1.1pp. The avirulent strain CU was least affected by competition with the virulent XL, and most adversely affected by competition with the avirulent 17XNL.

From these results we can infer that virulence is linked neither to competitive ability nor ‘fitness’ as measured through the ability of strains to transmit through mosquitoes in this species of malaria parasite, directly contradicting previous studies with *P. chabaudi* [7, 22], calling into question the validity of extrapolating general principles of the importance of within-host competition as a driver of the evolution of virulence from one parasite species to another. Malaria parasite species differ hugely in many important phenotypes, some of which, such as the timing of gametocytogenesis, will affect the evolutionary repercussions of within-host competition. Considering, for example, the cases of *P. falciparum* and *P. vivax*, the two most prevalent of the malaria parasites that infect humans, it may be reasonable to postulate that the evolution of *P. vivax* strains might be less influenced by within-host competition than *P. falciparum*, due to the former species’ propensity for producing gametocytes early on in infections [61], before the influence of inter-strain within-host competition would manifest.

In summary, previous experiments with *P. chabaudi* have appeared to show that within-host competition would drive the evolution of virulence; our results with *P. yoelii* contradict this, and this discrepancy is probably best explained by phenotypic differences between the species with respect to the timing of gametocyte production. We urge caution, therefore, when extrapolating the results of experiments dependent on variable phenotypic traits with one species of malaria parasite to any other.

The fact that our experiments with *P. yoelii* yield contrasting results to those performed with *P. chabaudi* highlights the importance of parasite biology when considering informative models for the evolution of various traits, including virulence. Extrapolation from one species to another is problematic when parasite biology varies greatly between species. Furthermore, as host-parasite interactions are of crucial importance in these types of studies, it should be emphasized that the rodent malaria parasites are not, naturally, parasites of *Mus musculus*, but rather of *Grammomys surdaster* and *Thamnomys rutilans* (Reviewed in [62]), and that the typical pathological outcomes of malaria parasite infections in these natural hosts is very different from that observed in laboratory mice. This point is illustrated further by studies showing that the outcome of within-host competition can be significantly different depending on the laboratory mouse strain used [59].

In summary, mixed strain infections of *P. yoelii* were found to cause more severe disease in mice than single infections of the constituent strains. There was no apparent increase in the infectivity of mixed infections to mosquitoes, and mixed infections did not result in greater oocyst burdens per infected mosquito. Within-host competition generally led to a reduction in parasite fitness, the degree of which varied between strains. Importantly, we found no evidence that virulent strains were more competitive than less virulent strains, and conclude that, in the case of *P. y. yoelii*, within-host competition would not lead to the selection of virulent strains.

## Materials and Methods

### Ethics statement

Laboratory animal experimentation was performed in strict accordance with the Japanese Humane Treatment and Management of Animals Law (Law No. 105 dated 19 October 1973 modified on 2 June 2006), and the Regulation on Animal Experimentation at Nagasaki University, Japan. The protocol was approved by the Institutional Animal Research Committee of Nagasaki University (permit: 1207261005–2).

### Parasites and hosts

We used four strains of rodent malaria parasite *Plasmodium yoelii yoelii*, three of which are phenotypically distinct lines that are isogenic except for polymorphisms at those loci that confer virulence [63]. These are *Plasmodium yoelii yoelii* 17XNL (wild-type, non-virulent) [64], *P. y. yoelii* 17XL (virulent) [64], and *P. y. yoelii* 17X1.1pp (intermediate virulence) [65], and a genetically unrelated strain *P. y. yoelii* CU, which is of wild-type, non-virulent phenotype [65]. Eight-week old female CBA mice (SLC Inc., Shizuoka, Japan) were housed at 26°C and fed on maintenance diet with 0.05% para-aminobenzoic acid (PABA)-supplemented water to assist with parasite growth. *Anopheles stephensi* mosquitoes, used in the transmission experiments, were housed in a temperature and humidity controlled insectary at 24°C and 70% humidity, adult flies being maintained on 10% glucose solution supplemented with 0.05% PABA.

### Virulence and competitive ability

To address the question of whether within-host competition leads to increased virulence, we infected groups of mice with either of the strains on their own or together with a competitor strain. Densities of each strain in mixed infection were monitored using strain-specific real-time quantitative PCR [59, 66], replication rates were measured by asexual parasitaemia and virulence was quantified through monitoring anaemia, live-weight loss [67] and host mortality.

Seven experimental groups of four mice each were set up to understand the effects of interactions between different parasite strains on the host and to compare the fitness of a strain in single versus mixed strain infections. Four of these groups were each singly-infected by i.v inoculation with CU, 17XNL, 17X1.1pp, or 17XL parasites (1 × 10^6^ parasitised erythrocytes in 0.1mL). The remaining three groups each received a total of 2 × 10^6^ parasites comprising a mixture of equal numbers of CU + 17XNL, CU + 17X1.1pp, and CU + *P. yoelii* 17XL. Inocula were prepared by taking blood from the tail vein of the donor mouse and diluting it in medium suitable for parasite maintenance (50% heat-inactivated foetal calf serum, 50% Ringer’s solution [27 mM KCl, 27 mM CaCI_2_, 0.15 M NaCI], with 20 units of heparin/ml mouse blood) to the appropriate concentration for the inoculum size. The requisite volume of blood was calculated from the blood cell density and parasitaemia in donor mice counted immediately before experimental sub-inoculations.

To accurately quantify the proportion of each strain used in the mixed strain infections, DNA was later extracted from a sample of each inoculum for real-time quantitative PCR (qPCR) analysis. Mouse red blood cell (RBC) densities and live-body weights were monitored as indicators of virulence. RBC densities were measured using a Coulter Counter (Beckman Coulter, Florida) from a 1:40,000 dilution of 2 μl sample of tail blood in Isoton (Beckman Coulter, Florida) solution. Parasite replication rate was assessed for 30 days by counting the proportion of RBCs infected by asexual parasites (parasitaemia) on Giemsa’s solution-stained thin blood smears from tail vein blood. Densities of gametocytes, the blood stage parasites that are transmissible to mosquitoes, were obtained by counting the number of RBCs containing mature gametocytes (distinguishable from asexual parasites by their morphology and presence of pigment as detected by polarized light) in the same thin blood smears used for counting asexual parasites. Asexual parasite density and gametocyte density were calculated from the product of RBC density and parasitaemia or gametocytaemia. Parasite densities in mixed strain infections were measured at specific time-points from day 1 to day 30 p.i using strain-specific qPCR from DNA prepared from 10-µl tail blood samples which were collected daily into physiological citrate saline solution, spun down and the pellet stored at −80°C prior to DNA extraction. The experiment was repeated once.

### Mosquito transmission

To assess whether the outcome of the within-host competition was a determinant of transmission success, we fed mosquitoes on mice infected with either single or mixed infections. Transmission was measured by density of sexual forms, gametocytes, in the blood, the proportion of mosquitoes infected after taking a blood-meal from the mouse, and the numbers of oocysts present on infected mosquito midguts. The number of oocysts produced and the proportions of mosquitoes that were infected were also used as indicators of vector fitness.

Twenty female *Anopeheles stephensi* mosquitoes (seven- to eleven-days post-emergence) were allowed to take blood meal from anaesthetised and immobilised mice in both single and mixed infections groups on day 3 p.i and the same was repeated with a fresh group of 20 mosquitoes day 4 p.i. Groups of mosquitoes that had fed on the same mouse were housed in individual pots. Seven to eight days post-feed, mosquitoes were immobilised and their midguts dissected to determine the number of oocysts and the percentage of oocyst-infected mosquitoes. To quantify the proportion of each strain in mixed-strain infections, mosquito midguts from the mixed-infection groups were suspended in PBS, spun down and the pellet stored at −80°C before DNA extraction and subsequent qPCR.

### DNA extraction and real time quantitative PCR (qPCR)

The proportion of co-infecting strains in mixed infections was determined by using qPCR measurement of the copy number of parasite’s MSP-1 gene. The MSP-1 gene, located on chromosome 8 [68] contains regions of high sequence polymorphism between clones that facilitate the design of allele-specific primers that can act as clone-specific genetic markers. DNA was extracted from infected mouse blood and infected mosquito midguts using EZ1 DNA investigator kit (Qiagen) according to manufacturer’s instruction. The extracted DNA was used for qPCR using the Power SYBR Green PCR kit (Applied Biosystems, UK) on a 7500 Real Time PCR system (Applied Biosystems, UK). Copy numbers of parasite *msp-1* were quantified with reference to a standard curve generated from known numbers of plasmids containing the same gene sequence. *Plasmodium yoelii* CU *msp1* and *P. yoelii* 17X *msp1* were amplified as previously described [69]. Description of the use of quantitative microsatellite markers to measure the proportions of parasites carrying markers linked to the putative genetic driver of virulence in mice and mosquitoes is given in S1 Text.

### Estimation of parasite fitness

Fitness coefficients were determined for the four strains based on the numbers of oocysts produced per mosquito that fed on mice infected with each of the strains on days 3 and 4 post-inoculation. The mean number of oocysts observed on the mid-guts of mosquitoes fed on mice with infections of the various strains averaged between days 3 and 4 were taken as an infectivity index. These were then standardized against the strain with the highest infectivity (CU), so that CU had a fitness coefficient of 1. In mixed infections, the proportion of each strain was determined by qPCR, and the “adjusted number of oocysts” calculated for each strain (number of oocysts multiplied by the strain frequency):

Fitness coefficient = mean number of oocysts per mosquito fed on strain X (day 3 + 4) / mean number of oocysts per mosquito fed on strain CU (day 3 + 4)

### Statistical analyses

All graphs were generated using GraphPad Prism (GraphPad Software Inc, USA). All statistical analyses were performed using R [36]. Detailed explanations of the statistical treatments used for each analysis are given in the relevant results section. All experiments were subject to full independent repeats with the exception of the experiment in which infectious mosquitoes were allowed to feed on naïve mice in order to measure whether parasite strain proportions present in mosquito oocysts were indicative of the proportions observed in mice following transmission, which were performed once.

## Supporting Information

S1 Fig Gametocyte density of single and mixed strain infections on day 3 (Panel A) and Day 4 (Panel B) post inoculation of mice with 1 × 10^6^ blood stage *Plasmodium yoelii yoelii* parasites. Error bars indicate the standard error of the mean for groups of 4 mice. Gametocyte density was calculated by multiplying gametocytaemia by red blood cell density.
(TIF)

S2 Fig In order to determine whether the parasite strain proportion measured in oocysts reflects the proportions inoculated into mice following the bites of infectious mosquitoes, we allowed five female mosquitoes to bite naïve mice 16 days after they were fed on infected mice. We then measured the proportions of genetic material from parental clones in the resulting infections. This analysis was performed using both qPCR (**Panels A, B and C**), measuring the proportions of the *msp1* gene, and microsatellite typing using a marker closely linked to the gene known to control virulence differences between these strains (**Panels D, E and F**). Bars indicate the proportion of each strain measured in individual mice. **Panels A** and **D** show proportions in mice prior to mosquito feeding. **Panels B** and **E** show the proportions of the strains measured in the oocysts of mosquitoes fed on those mice. **Panels C and F** show the proportions of strains measured in mice 5 days after being bitten by those mosquitoes.
(TIF)

S1 Text A description of the additional methodology used to generate S2 Fig. (DOCX)
