# Supplementary figures and images for: Within-host Competition Does Not Select for Virulence in Malaria Parasites; Studies with Plasmodium yoelii
Source: PLoS Pathog. 2015 Feb 6;11(2):e1004628. doi: 10.1371/journal.ppat.1004628 (PMC4450063; doi:10.1371/journal.ppat.1004628)

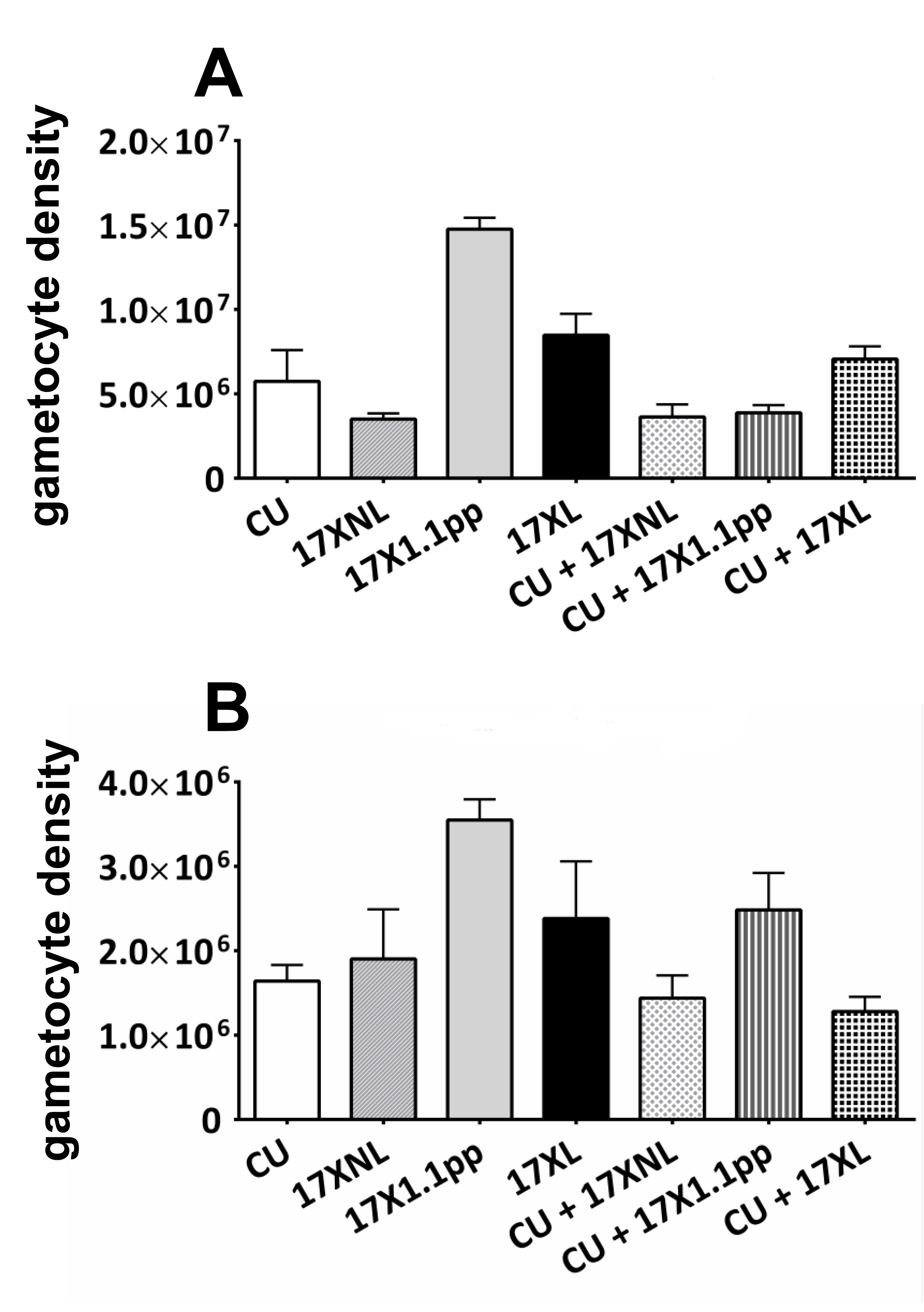

Supplement: S1 Fig — Error bars indicate the standard error of the mean for groups of 4 mice. Gametocyte density was calculated by multiplying gametocytaemia by red blood cell density. (TIF) [file ppat.1004628.s001.tif]

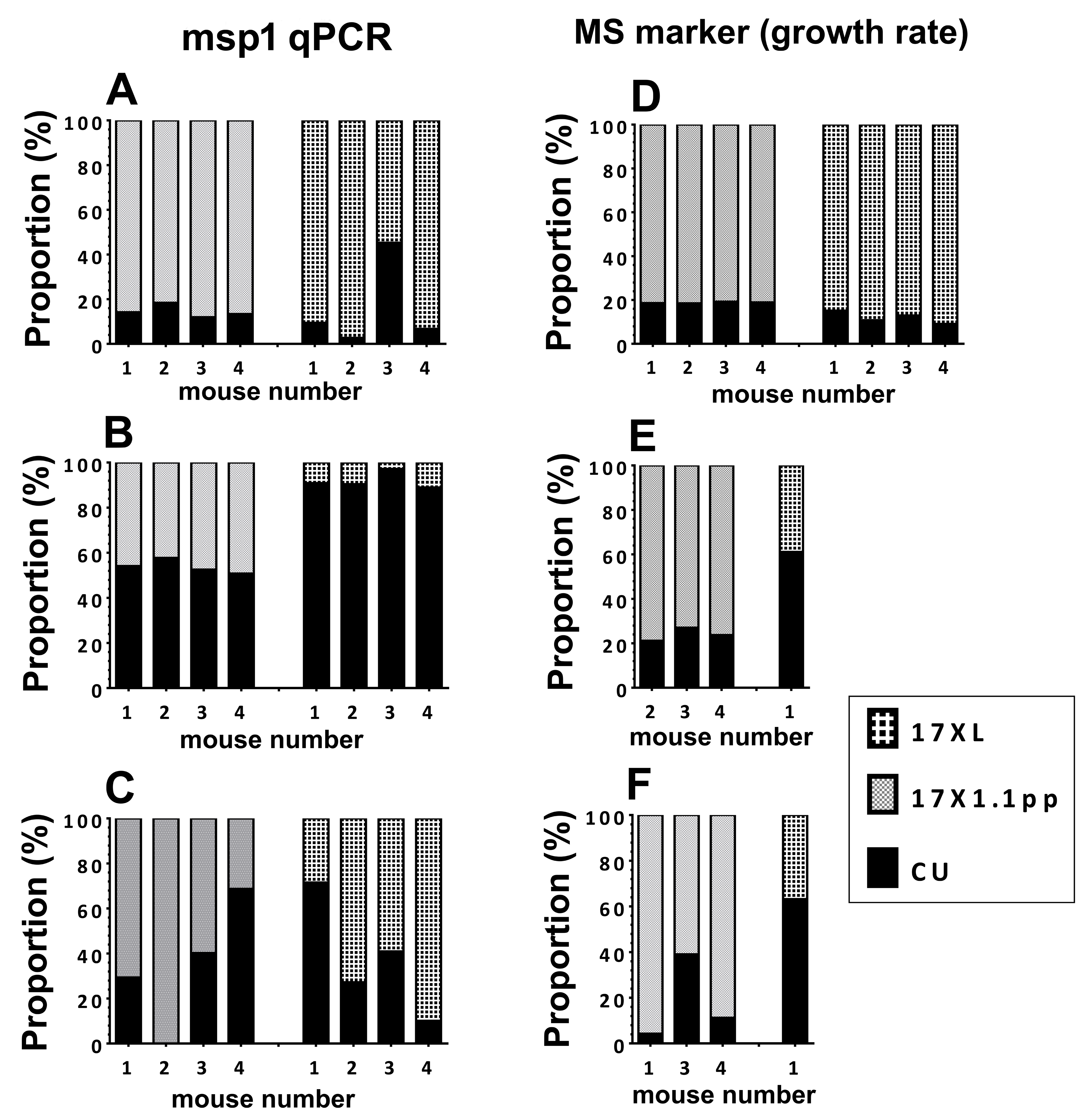

Supplement: S2 Fig — We then measured the proportions of genetic material from parental clones in the resulting infections. This analysis was performed using both qPCR (Panels A, B and C), measuring the proportions of the msp1 gene, and microsatellite typing using a marker closely linked to the gene known to control virulence differences between these strains (Panels D, E and F). Bars indicate the proportion of each strain measured in individual mice. Panels A and D show proportions in mice prior to mosquito feeding. Panels B and E show the proportions of the strains measured in the oocysts of mosquitoes fed on those mice. Panels C and F show the proportions of strains measured in mice 5 days after being bitten by those mosquitoes. (TIF) [file ppat.1004628.s002.tif]
