# Supplementary material for: Within-host Competition Does Not Select for Virulence in Malaria Parasites; Studies with Plasmodium yoelii
Source: PLoS Pathog. 2015 Feb 6;11(2):e1004628. doi: 10.1371/journal.ppat.1004628 (PMC4450063; doi:10.1371/journal.ppat.1004628)
Supplement: S1 Text — (DOCX) [file ppat.1004628.s003.docx]

**Supplementary Text S1**

**Supplementary Methods for figure S2**

**Microsatellite analysis:** To measure the proportion of parasite strains transmitted by mosquitoes harbouring mixed malaria parasites to a host, microsatellite analyses was performed on parasite gDNA extracted from blood stage infection, oocyst, and sporozoite-initiated infection. A locus containing a microsatellite marker closely linked to the gene known to control growth rate differences between these strains was amplified using a M13-tagged forward primer 5’CACGACGTTGTAAAACGACTGCCATATATGCAGAGCT3’, and a reverse primer 5’GTTATGGAGGATGTTAGTT3’ and a fluorescently labelled M13-Hex primer. PCR reactions were composed of 0.2μl of each of the three primers (all at 10μM concentration), 0.32μl of MgCl_2_, 0.8μl of 10×ExTaq buffer, 0.15μl of ExTaq polymerase (TaKaRa, Japan) and 1.5μl of extracted gDNA in a final reaction volume of 8.1μl. Reactions were run on an eppendorf MasterCycler gradient thermocycler with the following conditions: 94°C/2 min, 40×[94°C/20sec, 50°C/20sec, 60°C/20sec], 60°C/5min. Subsequently, 1.5μl of 40× dilution of PCR product was added to 8μl of formamide and 0.08μl of GeneScan™ 500 LIZ size standard. This solution was then heated at 95°C for 5 min before being processed in the ABI PRISM 3730 Genetic Analyzer. Products were analysed using GeneMapper Software. Microsatellite data was calibrated against qPCR measurements of artificially produced mixtures of parasite strains at known proportions. Artificial mixtures of blood containing known proportions of both clones were produced, and subjected to a qPCR assay. In order to produce the artificial mixtures, the number of parasites per μL of blood was determined for each of two mice, infected with either 17X1.1 or CU, by microscopy (counting the number of parasite nuclei per red blood cell (RBC)) and flow cytometry (to determine RBC density). A “proportion series” of 1:1, 1:4, 1:9, and 1:19 of CU: 17X1.1 was produced in this manner. This proportion series always included 17X1.1 at equal or higher proportions to CU, as the number of parasites per μL of blood was much larger for the 17X1.1 infected mouse than for the CU infected mouse, due to differences in growth rate between the clones. For this polymorphic microsatellite marker, the “proportion series” as quantified by qPCR was analysed by measuring the relative heights of peaks corresponding to the two clones.
